# Long-term outcomes of an internet-delivered cognitive enhancement intervention targeting residual cognitive deficits after major depressive disorder: a 2-year follow-up of an open trial

**DOI:** 10.3389/fpsyg.2023.1194689

**Published:** 2023-08-15

**Authors:** Sunniva Brurok Myklebost, Tine Nordgreen, Oda Stakkestad Klakegg, Åsa Hammar

**Affiliations:** ^1^Division of Psychiatry, Haukeland University Hospital, Bergen, Norway; ^2^Department of Biological and Medical Psychology, Faculty of Psychology, University of Bergen, Bergen, Norway; ^3^Department of Global Public Health and Primary Care, Faculty of Medicine, University of Bergen, Bergen, Norway; ^4^Department of Clinical Sciences, Faculty of Medicine, Lund University, Lund, Sweden; ^5^Office for Psychiatry and Habilitation, Psychiatry Research Skåne, Skåne, Sweden

**Keywords:** cognitive remediation, depression, digital, cognitive training, relapse

## Abstract

**Background:**

Cognitive deficits are common and disabling residual symptoms following major depressive disorder (MDD) and are related to increased risk of relapse. Residual cognitive deficits should thus be considered an important target for treatment. However, few have reported long-term outcomes of interventions targeting residual cognitive deficits.

**Objective:**

This study aimed to (1) investigate change between pre-treatment and 2-year follow-up assessments in cognitive deficits, rumination, and symptoms of MDD after an internet-delivered intervention targeting residual cognitive deficits; (2) to investigate stability in outcomes between 6-month and 2-year follow-up assessments; (3) to report the number of participants’ experiencing a new episode of MDD in the follow-up period; and (4) to investigate differences in outcomes between those who experienced a new episode of MDD and those who did not.

**Methods:**

A total of 43 partly remitted adults were included to test a guided internet-delivered intervention, which consisted of 10 modules involving psychoeducation, cognitive strategies, and attention training. Participants were assessed at pre-treatment, post-treatment, after 6-months, and after 2-years, with measures assessing self-reported residual cognitive deficits, rumination, symptoms of MDD and relapse. Overall, 32 participants completed the 2-year follow-up assessment.

**Results:**

Between the pre-treatment and 2-year follow-up assessments, there was a reduction in cognitive deficits and rumination, while there was an increase in symptoms of MDD. Cognitive deficits were stable between the 6-month and the 2-year follow-up, while there was an increase in rumination and symptoms of MDD. Thirteen of 32 participants reported a new episode of MDD during the follow-up period. The relapse group reported longer duration of MDD at pre-treatment and showed a difference in all outcomes after 2 years compared to the no-relapse group. The no-relapse group showed improvement in MDD symptoms at post-treatment, while the relapse group did not.

**Conclusion:**

Delivering cognitive enhancement interventions over the internet is potentially related to stable improvements in residual cognitive deficits. The effects on rumination and symptoms of MDD are less certain. Lack of improvement in MDD symptoms after the intervention period should be investigated as an indicator of relapse. Results should be interpreted with caution due to the lack of control group and sample size.

## 1. Introduction

Major depressive disorder (MDD) is a serious disorder that affects about 150 million individuals worldwide and is a leading cause of disease burden (World Health Organization, 2017). The disorder often takes a chronic course, as 50% is reported to experience a new episode of MDD during a 2 year period after receiving psychotherapeutic treatment, and the risk of relapse increase by 16% with each new episode (Solomon et al., 2000; Vittengl et al., 2007). The negative impact of MDD, can to some extent, be explained by cognitive deficits involving difficulties with attention, memory, and executive processes. Specifically, the experiencing of cognitive deficits is related to reduced occupational performance and psychosocial functioning (Kim et al., 2016; Saragoussi et al., 2018; Ott et al., 2019). In addition, during periods of remission, many remitted individuals report cognitive deficits as one of the most common and disabling residual symptoms (Conradi et al., 2011; McClintock et al., 2011; Christensen et al., 2020). Moreover, risk of relapse is also associated with experiencing residual cognitive deficits (Lorimer et al., 2020; Schmid and Hammar, 2021). Therefore, residual cognitive deficits are an important treatment target, but access to interventions developed to target and remediate residual cognitive deficits is still scarce. One explanation for the limited access to clinical interventions targeting cognitive deficits could be the lack of therapist resources and healthcare personnel with expert knowledge of cognitive deficits and treatment options (Medalia and Erlich, 2017), which could be solved by delivering interventions over the internet in order increase access to treatment and knowledge. Internet-delivered interventions targeting residual cognitive deficits have indeed been shown to be feasible, and the short-term clinical outcomes are promising (Hoorelbeke and Koster, 2017; Hammar et al., 2022; Myklebost et al., 2022c). However, there is still a need to explore the long-term clinical outcomes and preventive effects of internet-delivered interventions for residual cognitive deficits after MDD.

The main approaches in interventions to enhance cognition in mental health are drill-and-practice cognitive training aiming to restore cognitive functions, and strategy-based training to compensate for cognitive deficits (Bowie et al., 2020), as well as psychoeducation with regard to the impact and consequences of such deficits in daily life (Douglas et al., 2019). Efficacy of cognitive interventions are increased by providing support from an active and trained therapist guiding the use of cognitive strategies, in addition to including discussion groups and psychiatric rehabilitation addressing the recovery of daily life functioning (Lejeune et al., 2021; Vita et al., 2021).

Extensive research has been conducted on the benefits of cognitive interventions in individuals with schizophrenia (Vita et al., 2021), and among psychosocial interventions are cognitive remediation the treatment approach with the highest degree of recommendation (Vita et al., 2022). However, there is less research on interventions aiming to improve cognition in individuals with a history of MDD. Short-term effects of cognitive enhancement interventions in formerly depressed adults show promising results in improving self-reported cognitive deficits, performance in neuropsychological tests, rumination, and daily life functioning (Hoorelbeke and Koster, 2017; Listunova et al., 2020b; Hammar et al., 2022; Myklebost et al., 2022c; Vicent-Gil et al., 2022a). A recent meta-analysis of cognitive enhancement interventions that included samples both in the acute state of MDD and in remission found improved short-term performance for neuropsychological tests, functioning in daily life, and symptoms of MDD (Legemaat et al., 2021). However, these effects did not remain after 3 months, although a weakness associated with the results was the limited number of studies reporting long-term results.

There are several 6-month follow-up studies that have reported promising findings on cognitive enhancement interventions in MDD (Myklebost et al., 2022c; Vicent-Gil et al., 2022a). However, to our knowledge, only one previous study has investigated the 1-year follow-up results of cognitive enhancement interventions for individuals partly in remission from MDD (Hoorelbeke et al., 2021). This study showed that drill-and-practice cognitive training, compared to a low cognitive demand condition, was related to lower recurrence of depression after 1 year. Both groups did show change in self-reported residual cognitive deficits and rumination. In the acute state of MDD, a 2-year follow-up study by Hagen and Stubberud (2021) found that neither strategy-based goal management training nor drill-and-practice computerized cognitive training, were related to significant changes in self-reported cognitive deficits. They did, however, find significant improvement in symptoms of MDD and rumination. Taken together, research on long-term effects of cognitive enhancement interventions in adults remitted from MDD is scarce and inconclusive. There is also limited knowledge of the clinical course of those relapsing, which is of importance in order to tailor interventions to those individuals not gaining from the effects of treatment.

In the current study, we present follow-up results from an assessment 2 years after an open trial investigating the clinical outcomes of an internet-delivered intervention targeting residual cognitive deficits in partly remitted adults. The intervention was planned and developed based on qualitative interviews with formerly depressed adults and therapists (Myklebost et al., 2022a). The intervention was self-tailored and guided, and included the following key elements: attention training, compensatory strategy training, psychoeducation, and tasks targeting worrying and rumination. Results from the open trial showed large improvements in self-reported cognitive deficits and rumination, and symptoms of MDD did not deteriorate. The results were stable at the 6-month follow-up (Myklebost et al., 2022c).

The current study had four aims:

1. Investigate overall change in self-reported residual cognitive deficits, rumination, and symptoms of MDD between pre-treatment and 2-year follow-up assessments.
2. Investigate if clinical outcomes are stable between 6-month and 2-year follow-up assessments.
3. Describe participants’ reports of experiencing relapse of MDD between post-treatment and follow-up assessments.
4. Explore differences in clinical outcomes between individuals who relapsed compared to those who did not relapse.

## 2. Materials and methods

### 2.1. Design and procedures

The study was an open trial with pre-treatment, post-treatment, 6-month, and 2-year follow-up assessments. The study protocol was approved by the Regional Committee for Medical Research Ethics of Western Norway (2018/2384/REK vest).

The recruitment was conducted by means of national advertisements in social media, public posters, and newspapers. Individuals who were interested made contact for a brief telephone interview with a clinical psychologist or psychology student under supervision. For the assessment of symptoms of MDD the Montgomery-Åsberg Depression Rating Scale (MADRS; Montgomery and Åsberg, 1979) was used. The Norwegian M.I.N.I version, which is a structural clinical interview for psychiatric diagnoses in the DSM-IV (Leiknes et al., 1999), was applied to assess previous and current diagnosis of MDD. Further evaluation of eligibility was conducted online, and included the Montgomery-Åsberg Depression Rating Scale Self-Report (MADRS-S; Svanborg and Åsberg, 2001). Additionally, the participants were presented with open-ended questions such as “Do cognitive difficulties affect your daily life activities?” and “Describe your experience of cognitive difficulties.” A clinical psychologist evaluated the responses. Inclusion criteria were: (a) previously received treatment for MDD in primary or secondary healthcare services; (b) few or minor symptoms of MDD with no cardinal symptoms (reported sadness, loss of interest, and inability to feel, <16 MADRS-S); (c) self-reported residual cognitive symptoms that affect daily functioning; (d) no changes in psychopharmacological treatment status during the study period; (e) age between 18 and 65 years; and (f) internet access. The following exclusion criteria were used: (a) self-reported substance abuse; (b) neurological conditions or damage (e.g., autism, cerebral hemorrhage, or brain tumor); (c) bipolar disorder; and (d) psychosis.

### 2.2. Intervention

The intervention, called RestDep, was delivered over the internet. The intervention was created for adults who experience residual cognitive deficits after MDD. The intervention consisted of 10 modules. Core features of the intervention included: (1) general psychoeducation and tasks concerning residual cognitive deficits and rumination; (2) attention training; and (3) compensatory strategies. The intervention could be accessed using smartphones, tablets, or personal computers. Participants were encouraged to finish the program within 5–7 weeks, and to complete two modules each week. The intervention could be self-tailored as participants could choose strategies that they would like to implement and use in their everyday life. Selected strategies and training tasks were stored in a personal workbook, called “My plan,” where participants evaluated their usefulness. After completing the intervention, participants were asked to continue their cognitive training and use the strategies that they found useful. Included in the intervention was also a brief telephone guidance conversation, given weekly by a clinical psychologist or an advanced psychology student in clinical psychology, with the aim of increasing motivation and providing feedback on assignments. All therapists had at least 4 h of introduction to the intervention and received weekly supervision by a senior clinical psychologist. The intervention has been presented in detail elsewhere (Myklebost et al., 2022b,c).

### 2.3. Clinical outcomes

#### 2.3.1. Residual cognitive deficits

The Behavior Rating Inventory of Executive Function-Adult Version (BRIEF-A; Roth et al., 2005) is a 75-item self-report questionnaire that was used to assess subjective cognitive deficits. Participants are asked to rate the frequency of experienced executive problems as “never,” “sometimes,” or “often.” Responses are summed for each score, with higher scores indicating lower executive functioning. Results from the BRIEF-A are combined into a Global Executive Composite (GEC). In this study we analyzed raw scores due to the lack of Norwegian norms and the use of raw scores in reports from similar intervention studies (Hagen et al., 2020; Hammar et al., 2022). The BRIEF-A has been validated in adults and shows high internal consistency (Roth et al., 2005; Ciszewski et al., 2014). BRIEF-A was used in the pre-treatment, post-treatment, 6-month, and 2-year follow-up assessments.

The five-item Perceived Deficits Questionnaire-Depression (PDQ-5; Sullivan et al., 1990) is a brief questionnaire that measures subjective cognitive deficits. It covers problems with concentration, memory, and executive functioning. The items are rated on a scale from zero (never) to four (almost always). Higher scores indicate greater severity of perceived cognitive difficulties. The PDQ-5 was used in the pre-treatment, post-treatment, 6-month, and 2-year follow-up assessments.

#### 2.3.2. Rumination

The Rumination Response Scale (RRS; Treynor et al., 2003) is a frequently used questionnaire to assess rumination associated with depression. The RRS consists of 22 items and measures degree of ruminative responses to depressed mood. Every item is rated on a scale from one (almost never) to four (almost always). Higher scores indicate higher levels of rumination (Roelofs et al., 2006). There are three subscales within the RRS: depression-related thoughts (12 items), brooding (five items), and reflection (five items). Previous studies have demonstrated that the RRS is a valid and reliable measure (Roelofs et al., 2006; Johnson et al., 2008). The RRS was administered at pre-treatment, at post-treatment, 6-month, and 2-year follow-up assessments.

#### 2.3.3. Symptoms of MDD

The Montgomery-Åsberg Depression Rating Scale Self-Report (MADRS-S; Svanborg and Åsberg, 2001) is a questionnaire that measure symptoms of MDD experienced during the past 3 days. The MADRS-S consists of nine items where various symptoms are rated on a seven-point scale ranging from zero to six. A higher sum score reflects higher levels of symptoms. Previous studies have shown that online versions of the MADRS-S have acceptable internal consistency (Holländare et al., 2010). Participants completed the MADRS-S in the pre-treatment, post-treatment, 6-month, and 2-year follow-up assessments.

#### 2.3.4. Relapse of MDD

Relapse of MDD was measured by asking participants whether they had experienced a new episode of MDD since the post-treatment assessment.

### 2.4. Statistics

SPSS Statistics version 27 was used to analyze the data. The significance level used was *p* < 0.05. Change over time were analyzed using linear mixed models for repeated measures. Data from all participants provided at pre-treatment were included in the linear mixed model analysis, following the intention-to-treat principle. The linear mixed models were fitted with full information maximum likelihood estimation. Estimation of missing data from the observed data was based on the assumption of data missing at random. An independent sample *t*-test or Chi-Square Test of Independence was used to analyze demographic and clinical differences between the relapse and no-relapse group. An independent sample *t*-test or a Mann–Whitney U-test (for non-normal distributed data) were used to examine the differences between the two groups in the pre-treatment, post-treatment, 6-month, and 2-year follow-up assessments. Paired sample *t*-tests or a Wilcoxon-test (for non-normal distributed data) were used to analyze clinical changes in the no-relapse and relapse group between the pre-treatment and post-treatment assessments. Normality of data was assessed using a Shapiro–Wilk test. A Bonferroni test was used to correct for multiple tests (*p* < 0.001).

## 3. Results

Of those completing the 2-year follow-up, 19 participants answered not having experienced a new episode of MDD since the post-treatment assessment, while 13 participants reported having experienced a new episode of MDD. Participants in the relapse group had more often been depressed for a year or more compared to the no-relapse group. There were no groups differences in age, gender, civil status, education, or number of MDD episodes. Table 1 presents the participants’ pre-treatment characteristics.

**TABLE 1:** Pre-treatment characteristics of the sample and comparisons between the relapse and no-relapse group on demographic and clinical differences.

Variables	Total sample (*n* = 43)	Non-responders (*n* = 11)	Relapse (*n* = 13)	No-relapse (*n* = 19)	*p*
Age (mean, *SD*)	35.3 (13.04)	32.3 (13.05)	36.2 (12.99)	36.4 (13.50)	0.977
Female	79.1%	81.8%	76.9%	78.9%	0.892
Higher education	69.8%	63.6%	61.5%	78.9%	0.282
Partner	46.5%	0%	61.5%	63.2%	0.926
**Duration MDD**					
<1 year	23.3%	17.3%	0%	36.8%	0.013
>1 years	76.7%	72.7%	100%	63.2%	
**Episodes MDD**					
1 episode	27.9%	9.1%	23.1%	42.1%	0.266
2 or more episodes	72.1%	90.9%	76.9%	57.9%	

Duration MDD: overall duration of MDD in years before pre-treatment assessment; Episodes MDD: number of MDD episodes before pre-treatment assessment.

Table 2 presents the results from linear mixed model analyses including data from 43 participants at pre-treatment assessment, 37 participants at post-treatment assessment, 32 participants at 6-month follow-up assessment, and 32 participants at the 2-year follow-up assessment. The results show a significant decrease in the BRIEF-A, PDQ-5, and RRS between the pre-treatment and the 2-year follow-up assessments. Scores in the MADRS-S showed a significant increase from the pre-treatment to the follow-up assessment after 2 years. After the Bonferroni test, the BRIEF-A and PDQ were the only outcome measures that still had a significant decrease from pre-treatment and 2-year follow-up assessments.

**TABLE 2:** Results from the linear mixed model analyses.

Variables	Estimated changes (*SE*)	[95% CI]	*p*
**BRIEF-A**			
Pre-treatment to 2-year FU 6-month to 2-year FU	−17.4 (3.4) 2.5 (3.7)	[−24.2 to −10.6] [−4.7 to 9.8]	**0.001** 0.492
**PDQ-5**			
Pre-treatment to 2-year FU 6-month to 2-year FU	−4.0 (0.6) 0.7 (0.7)	[−5.2 to −2.7] [−0.6 to 2.1]	**0.001** 0.259
**RRS**			
Pre-treatment to 2-year FU 6-month to 2-year FU	−3.9 (1.8) 4.7 (1.9)	[−7.4 to −0.3] [0.9 to 8.5]	0.036 0.015
**MADRS-S**			
Pre-treatment to 2-year FU 6-month to 2-year FU	2.9 (1.1) 2.6 (1.2)	[0.7 to 5.2] [0.2 to 4.9]	0.009 0.036

Bold indicates if the *p*-value is significant after Bonferroni correction; FU, Follow-up; BRIEF-A, Behavior Rating Inventory of Executive Function-Adult version Global Executive Composite; PDQ-5, Perceived Deficit Questionnaire-Depression 5-item; RRS, Rumination Response Scale; MADRS-S, Montgomery-Åsberg Depression Rating Scale Self-Report.

The linear mixed model analyses did not show any significant changes between the 6-month follow-up and the 2-year follow-up assessments in the BRIEF-A and PDQ-5, while there was a significant increase in the RRS and MADRS-S. After the Bonferroni test, none of the outcome measures showed significant changes from the 6-month follow-up and 2-year follow-up.

Sub-group analyses did show that, in the pre-treatment assessments, there were no significant differences in the clinical outcomes between the relapse and no-relapse groups. At the 2-year follow-up, there were significant differences between the two groups in the BRIEF-A (*p* = < 0.001), PDQ-5 (*p* = 0.008), and RRS (*p* = 0.001). Results from the Shapiro–Wilk test did show that data from all these outcomes measures were normally distributed, although not for the MADRS-S. The Mann–Whitney U-test were therefore used to assess group differences in the MADRS-S scores, while independent sample *t*-test analyses were used to assess change in the other outcome measures. Significant differences in the MADRS-S were shown between the groups at post-treatment, 6-month follow-up, and 2-year follow-up assessments (*p* = 0.026, *p* = 0.016, *p* = < 0.001, respectively). Table 3 presents the differences in clinical outcomes between the relapse and no-relapse groups across the assessments. After the Bonferroni test, there were still significant differences between the relapse and no-relapse group in the BRIEF-A, RRS, and MADRS-S at the 2-year follow-up. At the 6-month follow-up, there was still a significant difference between the groups in the RRS after adjusting the significance level.

**TABLE 3:** Differences between the relapse and no-relapse groups in the clinical outcome measures at pre-treatment, post-treatment, 6-month, and 2-year follow-up assessments.

Measure		Pre-treatment		Post-treatment		6-months FU		2-year FU	
		**Mean, (*SD*)**	***p***	**Mean, (*SD*)**	***p***	**Mean, (*SD*)**	***p***	**Mean, (*SD*)**	***p***
BRIEF-A	No-relapse	128.6 (16.2)		107.7 (17.8)		103.2 (16.2)		102.1 (19.7)	
	Relapse	133.9 (17.1)	0.376	123.9 (29.7)	0.072	126.0 (31.5)	0.017	132.7 (27.3)	**0.001**
PDQ-5	No-relapse	10.6 (4.4)		5.2 (2.8)		4.6 (2.5)		4.8 (3.5)	
	Relapse	8.9 (3.6)	0.258	6.5 (5.8)	0.349	6.6 (3.8)	0.096	8.5 (3.8)	0.008
RRS	No-relapse	43.3 (12.3)		33.5 (9.8)		28.4 (5.5)		31.9 (8.5)	
	Relapse	43.9 (14.1)	0.889	37.4 (12.4)	0.346	41.4 (13.5)	**0.001**	48.6 (17.2)	**0.001**
MADRS-S	No-relapse	8.5 (2.9)		6.6 (3.6)		6.5 (5.4)		7.0 (5.1)	
	Relapse	8.9 (3.2)	0.705	11.5 (6.1)	0.026	12.4 (5.8)	0.016	17.8 (10.1)	**0.001**

Bold indicates if the *p*-value is significant after Bonferroni correction; FU, Follow-up; BRIEF-A, Behavior Rating Inventory of Executive Function-Adult version Global Executive Composite; PDQ-5, Perceived Deficit Questionnaire-Depression 5-items; RRS, Rumination Response Scale; MADRS-S, Montgomery-Åsberg Depression Rating Scale Self-Report.

Analyses showed that those in the no-relapse group had a significant improvement in the BRIEF-A, PDQ-5, RRS, and MADRS-S between the pre-treatment and post-treatment assessments (Table 4). The relapse group had significant improvement in the PDQ-5 and RRS, while there was no significant change in the BRIEF-A and MADRS-S between the pre-treatment and post-treatment assessments. Results from the Shapiro–Wilk test showed that data from all outcome measures were normally distributed except for the MADRS-S. The Wilcoxon-test was therefore used to assess change in the MADRS-S from pre-treatment to post-treatment assessments, while paired sample *t*-test analyses were used to assess change in the other outcome measures. After the Bonferroni test, the no-relapse group still had significant improvements between pre-treatment and post-treatment assessments in the BRIEF-A, PDQ-5, and RRS. The relapse group did not show any significant improvements concerning any of the outcome measures after the Bonferroni test.

**TABLE 4:** Change in clinical outcomes from pre-treatment to post-treatment assessment in the no-relapse and relapse group.

Variables		Change scores	
		**Mean (SD)**	***p***
BRIEF-A	No-relapse	−20.8 (−12.3)	**0.001**
	Relapse	−10.0 (−23.0)	0.089
PDQ-5	No-relapse	−5.4 (−4.4)	**0.001**
	Relapse	−2.4 (−3.5)	0.024
RRS	No-relapse	−9.7 (−7.3)	**0.001**
	Relapse	−6.0 (−7.4)	0.011
MADRS-S	No-relapse	−1.9 (−3.0)	0.018
	Relapse	2.5 (6.9)	0.506

Bold indicates if the *p*-value is significant after Bonferroni correction; BRIEF-A, Behavior Rating Inventory of Executive Function-Adult version Global Executive Composite; PDQ-5, Perceived Deficit Questionnaire-Depression 5-items; RRS, Rumination Response Scale; MADRS-S, Montgomery-Åsberg Depression Rating Scale Self-Report.

## 4. Discussion

The aims of the current study were to investigate overall change in clinical outcomes between pre-treatment and follow-up assessments after 2 years, and stability in clinical outcomes between 6-month and 2-year follow-up assessments. An additional aim was, to report relapse rates for MDD and change in clinical outcomes between participants who had or had not experienced relapse of MDD. There was an overall decrease in the level of self-reported residual cognitive deficits and rumination between pre-treatment and 2-year follow-up assessments, while there was an increase in symptoms of MDD. Levels of self-reported residual cognitive deficits were stable between the 6-month and 2-year follow-up assessments, while there was an increase in rumination and symptoms of MDD. Of the 32 participants completing the 2-year assessment did 13 report a relapse of MDD. Participants in the relapse group had more often been depressed for a year or more at pre-treatment compared to the no-relapse group. Sub-group analyses showed significant mean differences in the clinical outcomes between the relapse and no-relapse groups in the 2-year follow-up assessments. Individuals in the no-relapse group showed improvement in symptoms of MDD from pre-treatment to post-treatment assessments.

The current study found that participants receiving the internet-delivered cognitive enhancement intervention did show reductions in self-reported residual cognitive deficits between the pre-treatment and follow-up assessments after 2 years. These improvements were stable between the 6-month and 2-year follow-up assessments. The results indicate that participants experienced improved abilities to manage cognitive deficits in daily life. This aligns with a 1-year follow-up study of remitted adults receiving a drill-and-practice cognitive enhancement intervention, which showed long-term improvement in self-reported residual cognitive deficits (Hoorelbeke et al., 2021). Contrasting findings were reported in a 2-year follow-up study that tested a strategy-based cognitive intervention in adults with MDD (Hagen and Stubberud, 2021). However, the 1-year follow-up study did not find significant differences between the those receiving the drill-and-practice intervention and the control group receiving a low cognitive load intervention. This illustrates the importance of including control groups to assess the efficacy of cognitive enhancement interventions. The lack of control group in the current study therefore warrants that the results be interpreted with caution. Moreover, none of the above studies have separately tested specific intervention components, such as psychoeducation, drill-and-practice training, and strategies. However, a randomized controlled trial with a 6-month follow-up did find that functional and drill-and-practice training combined with psychoeducation yielded better functional outcomes than psychoeducation alone (Vicent-Gil et al., 2022a). Long-term follow-up studies should therefore further explore the efficacy of intervention components and mechanisms of change to refine cognitive enhancement interventions. The current study explored the outcomes of the intervention using a measure developed to assess executive functioning in line with other long-term studies in the research field (Hagen and Stubberud, 2021; Hoorelbeke et al., 2021). Indeed, perceived executive functioning is important for functioning well in everyday life (Vicent-Gil et al., 2022b). However, to understand cognitive enhancement interventions’ efficacy on all aspects of residual cognitive deficits there is also a need to investigate the long-term effects on other subjective cognitive domains, such as attention and memory. This could be achieved by employing broad measures of cognitive functioning.

The results from the current study showed an overall reduction in rumination. However, the results were not stable, as we did find an increase in rumination between the 6-month and 2-year follow-up assessments. The elevated levels of rumination after the 6-month assessment might be related to the observed increase in symptoms of MDD. These findings are in contrast both with a previous study that reported long-term improvements in rumination and symptoms of MDD in adults with MDD receiving a strategy based intervention (Hagen and Stubberud, 2021) and with a randomized controlled trial (Vicent-Gil et al., 2022a) and meta-analysis (Legemaat et al., 2021) showing that cognitive enhancement interventions did not affect symptoms of MDD. Taken together, the mixed findings in the research literature illustrates the importance of further exploring the impact that cognitive enhancement interventions have on rumination and symptoms of MDD.

At the 2-year follow-up 13 (41%) of 32 participants reported having experienced a relapse of MDD. This number is relatively high compared to Hoorelbeke et al. (2021) who reported that 26% of their sample had a relapse during the year after having completed a cognitive enhancement intervention for remitted adults. However, it is likely that the relapse rate would have been higher if they had conducted a 2-year follow-up. The findings from the current study are, however, promising considering that 50% of individuals receiving psychotherapeutic treatment suffer a relapse after 2 years (Vittengl et al., 2007). Relapse rates for those only receiving medical treatment for MDD are even higher (Hollon et al., 2005). Moreover, many of the participants in the current study had experienced several episodes of MDD. Relapse rates for our sample could therefore be expected to be even higher, as research shows number of episodes being related to increasing risk of relapse (Solomon et al., 2000). Overall, research on the effects of cognitive enhancement interventions on preventing relapse of MDD should be explored further, as the evidence is still limited and inconclusive.

Sub-group analyses showed that there were no differences in the clinical outcome measures between the relapse and no-relapse groups at pre-treatment assessment. At post-treatment assessment, however, the relapse group had significantly higher levels of MDD symptoms compared to the no-relapse group. Moreover, the no-relapse group also had a significant improvement in symptoms of MDD, while the relapse group did not. At the follow-up assessments after 2 years, there were significant differences between the groups in residual cognitive deficits, rumination, and symptoms of MDD. These results show that those who did not relapse experienced improved symptoms of MDD after receiving the intervention, and over time they report lower levels of rumination and self-reported cognitive deficits. In contrast, at the 2-year follow-up the relapse group reported higher levels of MDD symptoms and rumination compared to pre-treatment levels, while the levels of residual cognitive deficits were similar to the pre-treatment levels. These results are in line with research suggesting that symptoms of MDD influence rumination and cognition (Olatunji et al., 2013; Miskowiak et al., 2016b). This implies that, in order to prevent a relapse of MDD, it might be relevant to provide booster sessions with intervention elements, such as cognitive behavior therapy, to target symptoms of MDD in the follow-up period for those not showing improvement in symptoms of MDD.

The demographic characteristics of those who do and do not experience a relapse of MDD could also provide indications of who is at risk of experiencing a new episode of MDD. In the relapse group, there were more participants with a severe history of MDD, such as a longer duration of MDD. Being depressed for a longer period might therefore have contributed to the relapse of participants in this study. This is in line with a previous study which found that those experiencing MDD for a year or less at pre-treatment reported larger improvements in residual cognitive deficits 6-months after receiving the current intervention than those with a longer duration of MDD (Myklebost et al., 2022b). Similarly, a randomized controlled trial reported duration of MDD to predict treatment response in partly remitted adults (Listunova et al., 2020a). These findings could be explained by participants with longer illness duration having more negative experiences with depression treatment and episodes with cognitive failures which might have affected their motivation to continue using strategies to cope with cognitive difficulties in everyday life. Moreover, some participants in the relapse group might have been currently depressed. Research shows that depression symptoms relate to negative self-perceptions of cognitive resources (Serra-Blasco et al., 2019). From this one might speculate if those in the relapse group experienced reduced initiative to make use of their cognitive resources and therefore does not cope with everyday life challenges, leading to negative self-representations, and increase in mood symptoms. However, these explanations are mere speculations. Nevertheless, the findings of the current study indicate that identifying those with a history of longer MDD duration before treatment could be of importance to tailor interventions. Future studies should explore these relationships further. Surprisingly, there were no differences between the groups regarding experiences of one or several episodes of MDD. The length of participants’ previous episodes of MDD might, however, have varied considerably. Moreover, analyzing the exact number of previous MDD episodes might have provided other outcomes besides the categorical approach employed in this study.

### 4.1. Strengths, limitations, and suggestions for future research

One of the strengths of this study is the long-term follow-up assessment, which provides more knowledge about the outcomes of internet-delivered cognitive enhancement interventions over time. Another strength is the investigation of outcomes between those who experienced a new episode of MDD and those who did not.

The main limitations of this study are the lack of control group and low sample size. Change in outcomes might therefore be a consequence of other factors such as participants spontaneous recovery, regressing to the mean, and false-positive errors (Cuijpers and Cristea, 2015). Caution is therefore warranted when interpreting the results. These limitations also apply to the research field in general as there is a need for more large-scaled randomized controlled trials to generate knowledge of the long-term efficacy of interventions targeting residual cognitive deficits after MDD. Currently, this is being addressed in an ongoing randomized controlled trial being conducted by our research group (ClinicalTrials.gov: 04864353). Conducting many analyses on small samples has been a common approach in this research field that also increases the risk for false-positive errors. This can be addressed by defining primary outcomes, and correcting for multiple analyses (Miskowiak et al., 2016a). In the current study, there was no pre-defined primary long-term outcome measure, although residual cognitive deficits measured by the BRIEF-A were pre-defined as a short-term outcome measure in the main trial (Myklebost et al., 2022c). Moreover, some of the results of this study are uncertain as they were not significant after correcting for multiple analyses. Consequently, these findings must be interpreted with caution. However, adjusting the significance level may increase the risk of making a type II error in explorative studies with small sample sizes. Another limitation is not assessing for potential confounding variables such as co-morbidity and the use of pharmacological treatments in the follow-up period, which could have implications for a change in perceived cognitive deficits (Miskowiak et al., 2017). Controlling for the use of pharmacological treatments in the follow-up period should therefore be considered in future trials investigating long-term outcomes.

Several relevant outcome measures were not included in the current study. Not administering neuropsychological assessments is a shortcoming as such measures provide insight into changes in objective cognitive deficits. In remitted and partly remitted adults, a lack of correlation is found between performance on neuropsychological tests and self-reported cognitive deficits (Serra-Blasco et al., 2019). Results from this study may therefore not be transferable to objective cognitive deficits, and not assessing objective measures of cognition might accordingly limit the validity of the results. Other limitations concerning outcome measures are not assessing the functional benefits of the intervention, such as work and social functioning. Research on interventions targeting cognitive deficits is often criticized for not investigating functional outcomes, which may be the most important and relevant outcome for the target group. Only a few studies targeted functional recovery specifically (Listunova et al., 2020b; Vicent-Gil et al., 2022a), showing promising results. Future research should therefore further investigate measures of functional outcomes. Neither neural responses to the intervention were explored, which may have given insights into the mechanisms of change. More research employing neuroimaging is needed to assess the functional and structural changes of interventions targeting residual cognitive deficits.

Lastly, several of the participants did not respond to the intervention and had a relapse. Overall, the intervention seemed to provide more improvement in self-reported residual cognitive deficits than rumination and symptoms of MDD. This may indicate a need to find other approaches to preventing relapse such as specifically targeting hot-cognition and emotional information processing.

## 5. Conclusion

This 2-year follow-up study shows that the use of the internet-delivered cognitive enhancement intervention was related to an overall and stable improvement of self-reported residual cognitive deficits after MDD, while the stability of effects on rumination and symptoms of MDD is less certain. The duration of previous episodes of MDD might represent a pre-treatment risk factor for relapse. The absence of improvement in symptoms of MDD after receiving internet-delivered cognitive enhancement interventions should be investigated further as an indicator of experiencing a new episode of MDD over time. Overall, the results from this study should be interpreted with caution due to the lack of control group, small sample size, and lack of objective measures of cognition.

## Data availability statement

The raw data supporting the conclusions of this article will be made available by the authors, without undue reservation.

## Ethics statement

The studies involving humans were approved by the Regional Committee for Medical Research Ethics of Western Norway. The studies were conducted in accordance with the local legislation and institutional requirements. The participants provided their written informed consent to participate in this study.

## Author contributions

SBM collected the data and wrote the main manuscript draft. OSK contributed to the abstract and method section. SBM, OSK, and ÅH participated in the analyses and interpretation of the data. TN and ÅH contributed with revisions of the draft manuscript. SBM, TN, and ÅH contributed with a conceptualization of the original trial. All authors contributed to the article and approved the submitted version.
